# Evaluation of a Digital Consultation and Self-Care Advice Tool in Primary Care: A Multi-Methods Study

**DOI:** 10.3390/ijerph15050896

**Published:** 2018-05-02

**Authors:** Julie Cowie, Eileen Calveley, Gillian Bowers, John Bowers

**Affiliations:** 1 Faculty of Health Sciences and Sport, University of Stirling, Stirling FK9 4LA, UK; eileen.calveley@stir.ac.uk (E.C.); j.a.bowers@stir.ac.uk (J.B.); 2 Adam Smith Business School, University of Glasgow, Glasgow G12 8QQ, UK; gillian.bowers@glasgow.ac.uk

**Keywords:** digital health, primary care, self-management, implementation

## Abstract

Digital services are often regarded as a solution to the growing demands on primary care services. Provision of a tool offering advice to support self-management as well as the ability to digitally consult with a General Practitioner (GP) has the potential to alleviate some of the pressure on primary care. This paper reports on a Phase II, 6-month evaluation of eConsult, a web-based triage and consultation system that was piloted across 11 GP practices across Scotland. Through a multi-method approach the evaluation explored eConsult use across practices, exposing both barriers and facilitators to its adoption. Findings suggest that expectations that eConsult would offer an additional and alternative method of accessing GP services were largely met. However, there is less certainty that it has fulfilled expectations of promoting self-help. In addition, low uptake meant that evaluation of current effectiveness was difficult for practices to quantify. The presence of an eConsult champion(s) within the practice was seen to be a significant factor in ensuring successful integration of the tool. A lack of patient and staff engagement, insufficient support and lack of protocols around processes were seen as barriers to its success.

## 1. Introduction

Across the United Kingdom (UK), the continued increase in patients requiring a General Practitioner (GP) consultation for non-urgent conditions is putting enormous strain on practices, with excessive demand for appointments and increased patient dissatisfaction with the service [1]. The situation is unlikely to improve with an increasingly elderly population worldwide, who are both living longer and with multi-morbidities [2].

Strategy documents such as the Modern Outpatient [3] advocate for a new model in care provision, where, given appropriate tools and resources, individuals are encouraged to take responsibility for their own health and well-being, adopting a self-management model. Such a shift in the balance of care attempts to promote wellness and in turn, reduce interactions with health care services [4]. There are many ways in which individuals can be encouraged to self-manage. One area that has received much attention is the use of eHealth, particularly in relation to use of websites and/or apps providing information on aspects such as illness trajectories, symptoms, and treatments relating to specific conditions [5,6].

In relation to primary care, the provision of self-management tools and online consultation have been promoted as ways to ease pressure on primary care services [7]. In addition, the potential for such technology to offer a more timely and convenient method of accessing outpatient services is recognised [3]. However, uptake of such tools by primary care has been slow and eHealth use in this area is still in its infancy [8]. As identified by Banks et al. [9], there are still limited research findings regarding the effectiveness of such tools [10] and there are only a minority of evaluations conducted within the UK [11]. It is imperative that such tools are fully evaluated before potential adoption and large-scale use.

Two prominent online self-management and consultation services in use across the UK are askmyGP (askmygp.uk) and eConsult (econsult.net). This research focuses on eConsult and its use across GP practices in Scotland.

eConsult aims to deliver safe and effective patient care by providing patients with a means for self-care advice. It was developed by the Hurley Group, which is a National Health Service (NHS) Partnership led by practicing GPs [12]. Accessed via the local GP surgery webpage, the tool provides individuals with: self-care assessment and advice in the form of symptom checkers, videos and self-help guides about the commonest conditions seen in general practice; triage of their circumstances in order to enable sign-posting to alternative services, such as community pharmacy and online counselling; access to NHS 24 (nhs24.scot) (national self-care and self-help advice for Scotland), access to 24/7 phone advice within one hour from NHS24 by requesting a call back through use of a web form; and consultations based on submission of a condition-based questionnaire by the patient with a response from the practice by telephone within one working day (an eConsult). Further details of eConsult are available at its website (econsult.net). 

eConsult has successfully been piloted and evaluated in England [9,11], but this is the first evaluation of the tool in Scotland. In this paper we discuss a Phase II trial of eConsult across 11 GP practices in Scotland. The paper discusses and reflects on these experiences and proposes recommendations for future roll-out of electronic self-care and consultation tools.

## 2. Materials and Methods

The key aims of the evaluation as agreed with the Scottish Government were to examine the impact of eConsult on patients; the impact of eConsult on the GP surgery and its staff; the time implications of eConsult; the cost/effectiveness of eConsult use; and identify barriers and facilitators to future implementation.

The evaluation used a multi-methods design [13] incorporating both quantitative data (log data from eConsult use, patient survey data) and qualitative data (interviews with GP staff and free text elements within patient survey). Use of this methodology is considered appropriate for evaluation of digital systems and has been used throughout the literature [14,15,16]. The qualitative elements of the design were employed to provide detailed data around eConsult perceptions and experiences, whilst the quantitative data provided factual information on eConsult use. Semi-structured interviews were used as these were felt appropriate for eliciting in-depth information around experience and use of eConsult, in addition to thoughts around the embedding of the system into the GP surgery environment [17]. The interviews were carried out by experienced qualitative researchers, who in addition to audio-recording, kept detailed field notes in order to enhance the validity and relevance of the research conducted [15].

A health economics analysis was conducted to elicit information regarding cost/effectiveness and time savings associated with the use of eConsult. Eleven GP practices across four Scottish NHS boards participated in the study. These had been selected on the basis of willingness to participate and to provide a mix of urban/ remote and rural practices.

### 2.1. Qualitative Data

#### 2.1.1. Sampling and Interviews/Focus Groups

Practice managers were contacted by the research team via telephone at least one month in advance of the site visit to discuss the evaluation and practicalities of meeting with practice staff. Practice managers at this point were given the option for staff to be involved in individual interviews with researchers or take part in a focus group (depending on what was most convenient for the practice). Practice staff were purposefully sampled via the practice manager who identified and contacted staff who were involved in the introduction of and/or daily use of eConsult. Participants were selected to represent the range of roles that were involved in the introduction of eConsult to the practice and/or the different stages of the eConsult process. Typically, GPs, practice managers, receptionists and administrators participated. Participant information sheets were shared with potential interviewees/focus group participants prior to the site visit, and full informed consent gained from all participants. Researchers used an interview topic guide that explored key themes around staff perceptions of patients’ view of eConsult, the impact of eConsult on the practice, and their own personal views on eConsult. Interviews and focus groups were conducted face-to-face and lasted between 25 and 50 min.

#### 2.1.2. Data Analysis

Interviews were audio-recorded, transcribed, anonymised and checked for accuracy prior to analysis. Data were analysed using a thematic framework approach [18] supported by NVivo 10. The initial framework was devised both inductively and deductively, using a small subset of interviews (*n* = 3) and the initial research questions of the study as agreed with the Scottish Government. The resulting framework continued to evolve through multiple rounds of analysis. Data were mapped and charted using diagrams and matrices by two research team members. Evidence of data that did not fit emerging patterns, and rival explanations for the data were actively looked for. Wider sense checking took place through regularly sharing emerging analysis with members of the wider research team, including the project’s Advisory Board. Use of framework analysis and applying the above approaches ensured high-level rigor and transparency was maintained throughout data analysis [19].

### 2.2. Quantitative Data

Quantitative data was collected from patient surveys (Appendix A) and eConsult log data over a 5-month period from 17 April to 17 August. The log data was collected from all patient interactions with the eConsult website in the participating practices over the designated period. Counts of the number of website visits, unique site visits in a 24-h period, site visits which were diverted to the urgent and emergency web page/phone line and eConsults submitted were recorded. In addition, demographic data on GP practices participating in the study was obtained from Information Services Division (ISD) Scotland [20,21].

Completion of the patient survey was voluntary for all patients who submitted an eConsult. Data from the survey was collected by the Hurley Group as part of their regular monitoring of service use. The data was provided to the research team at summary level, with no identifying information other than the source practice. Microsoft EXCEL was used to support the analysis of data.

### 2.3. Health Economics Analysis

Data around eConsult process and timings collected during the interviews and focus groups was used for the economic analysis. In addition, practices also provided data on their appointment time allocations for face-to-face and telephone consultations. Many of the timings for administrative tasks were estimates made by interviewees, hence some sensitivity analysis was undertaken using longer and shorter timings. This had little effect on the overall results as the variation in administrative cost was low in comparison to the cost of the GP input to the eConsult process. The cost of eConsult per patient on the practice roll was provided by the Hurley group.

**Figure 1:**
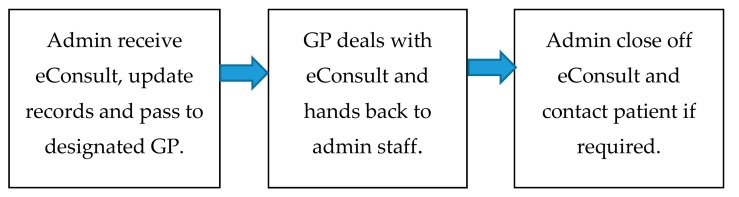
Simplified eConsult process.

Figure 1 gives a simplified view of the steps involved in processing an eConsult. The process assumed no triage (as this was not performed in most practices) and all eConsults were passed to the GP (though some might have been categorised as requiring administrative attention only). In most cases, only GPs dealt with non-admin eConsults, though some could have been processed by other GP health professional staff, for example an advanced nurse practitioner.

Future scenarios were devised from information collected in the interviews. In the interviews GPs were able to identify the types of eConsult that would be unlikely to require the patient to be recalled. The likely costs and savings from each scenario were calculated and adjusted to take account of findings from the English pilot study which found that for 8% of eConsults submitted the patient would not otherwise have attempted to consult their GP [11].

Ethical approval for the project was granted by the University of Stirling General University Ethics Committee on 6 April 2017. University ethics code: NICR 16/17 Chair’s action No.006.pa.

## 3. Results

Eleven GP practices across four Scottish NHS boards participated in the evaluation. GP practices were spread across a mix of urban/rural areas. One urban practice served a predominantly younger age group (50.5% under 25) and one rural practice had a slightly older age profile (28.1% aged 65+), while most had a reasonably evenly spread age distribution. Two practices had a high proportion of registered patients residing in the most deprived (bottom 15%) areas of Scotland, as defined by their Scottish Index of Multiple Deprivation (SIMD) codes [22]. Semi-structured interviews (*n* = 44) and a focus group (1 group of 4 staff) were used to collect qualitative data, along with analysis of free-text comments from patient surveys (*n* = 291, 6.5% response rate). The complement and number of staff at each practice are detailed in Table 1 below.

### 3.1. Impact of eConsult on Patients

The eConsult log data provided details of all eConsult website activity and eConsults submitted for each participating practice during the 5-month period from April to August 2017. Demographic data of eConsult users was not available for the trial period, but this was routinely collected from September 2017. This was supplemented with demographic data on GP practices in Scotland [20,21].

#### 3.1.1. eConsult Use by Patients

**Figure 2:**
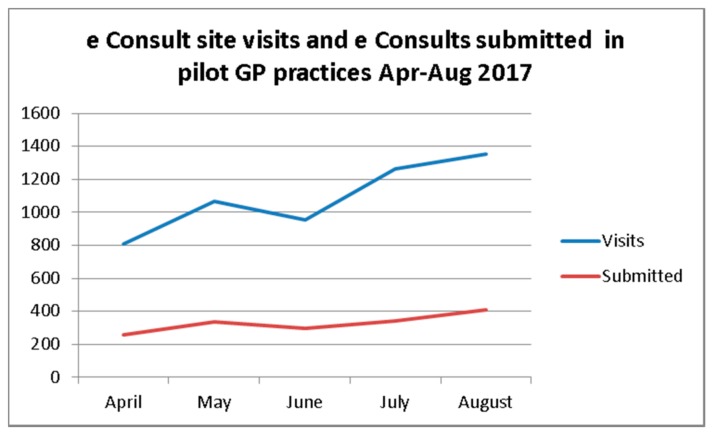
Comparison of eConsult site visits with eConsults submitted (April–August 2017).

From the eConsult log data shown in Figure 2, it is evident that interest and use of eConsult increased over the 5-month evaluation period. These five months of data were used to establish likely annual usage.

**Figure 3:**
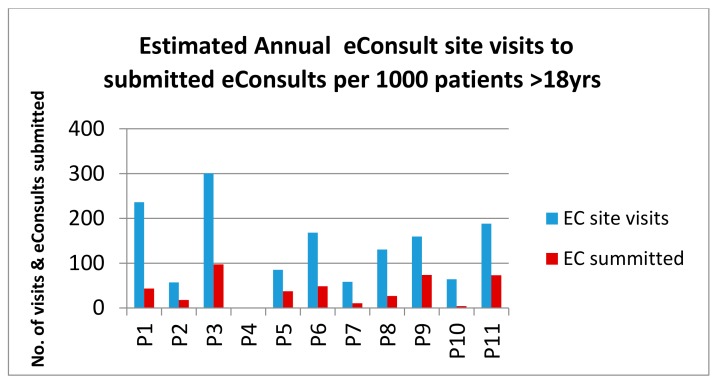
Estimated annual eConsult site visits and submissions per 1000 patients (age > 18 years *). * estimated number of patients aged 18+ from Information Services Division (ISD) count of patients aged 15–24 [20].

**Figure 4:**
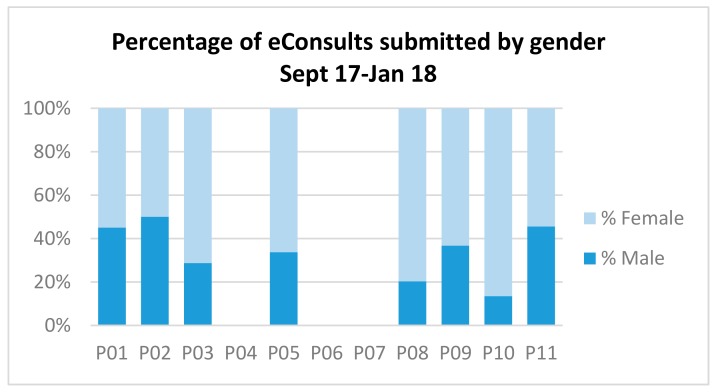
Percentage of eConsults submitted by gender 17 September–18 January.

Figure 3 shows the variation in usage at the different pilot practices. P4 did not participate for all of the trial period, hence that data is not shown in Figures 3, 4, 7–9. The highest usage was at practice P3 with a submission rate of almost 0.1 per patient per year. Very low submission rates were recorded at practices P7 and P10. The conversion rate from a site visit to an eConsult submission was variable, with practices P5, P9 and P11 recording around 40% of site visits resulting in a submission (46%, 39% and 44% respectively). The average conversion rate over all the pilot sites was around 30%. This may reflect marketing differences, where practices with the higher conversion rates promoted eConsult as a means of accessing the GP and those with lower rates might have stressed the use of the site as a means of self-help.

The distribution types of eConsult submitted were 32% specific conditions, 27% administrative help, 41% general advice (24% for a new problem, 17% for an existing problem). This mirrors findings from the English pilot of eConsult [11]. eConsults for specific conditions were distributed across a wide range of conditions with no particular trends emerging.

#### 3.1.2. Gender Profile in the eConsult Trial

Patients registered with GP practices in Scotland comprise of 49.5% Male/50.5% female. The trial practices were comprised of 49.6% Male/50.4% female. Within the practices the lowest male percentage was 45.9% (P8) and the highest 51.9% (P7). These were respectively a practice serving a large student population and a remote and a rural practice. 

Subsequently, 5 months of demographic data were analysed for the trial practices (17 September–18 January) This showed that 67% of eConsult users were female as compared with 60% of GP consultations for female patients [23]. There was some variation in the percentage of eConsults submitted by gender between the practices, this is shown in Figure 4. In all practices, the majority of eConsult users were female. No results are recorded for practices P4, P6 and P7 as they were no longer participating in the trial by January 2018.

#### 3.1.3. Age Profile in the eConsult Trial

**Figure 5:**
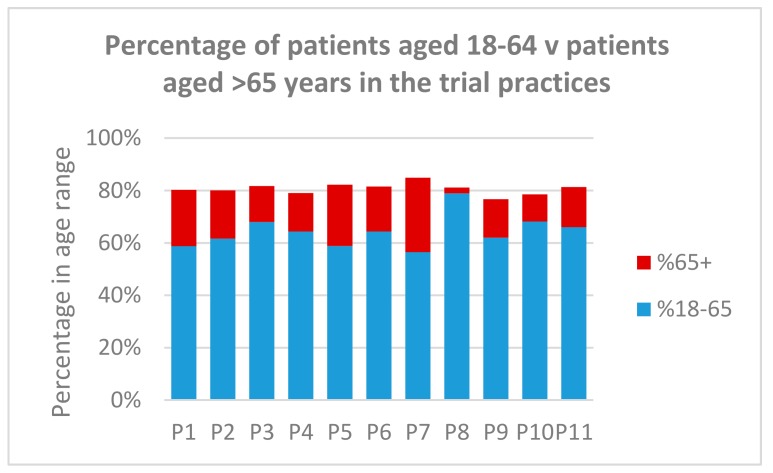
Comparison of the percentage of patients aged 18–64 with those aged over 65 years.

Figure 5 shows the age distribution of patients in the trial practices on the practice roll. The average across the practices was 64% of patients in the 18–64 age group and 20% in the over-65 age group. Notable deviations were P7 with 28.4% over 65 and P8 with 2.3% over 65 (this practice had a large number of students registered).

**Figure 6:**
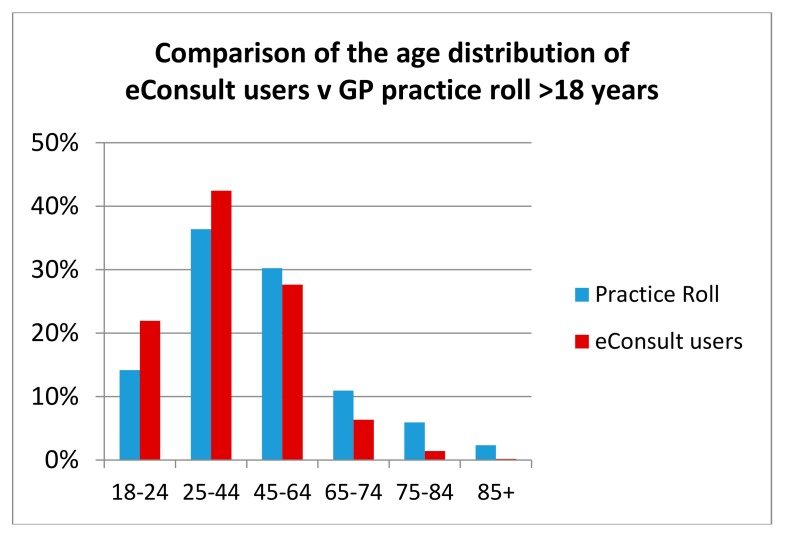
Comparison of the relative age distribution of patients aged over 18 with the age distribution of eConsult users in the trial practices (excluding P04, P06 and P07).

Figure 6 compares the percentage age distribution of eConsult users with the percentage age distribution of patients in the trial practices aged over 18 years. It can clearly be seen that a greater proportion of patients in the younger age groups (18–44) used eConsult than in older age groups. This finding concurs with that of other studies [24].

**Figure 7:**
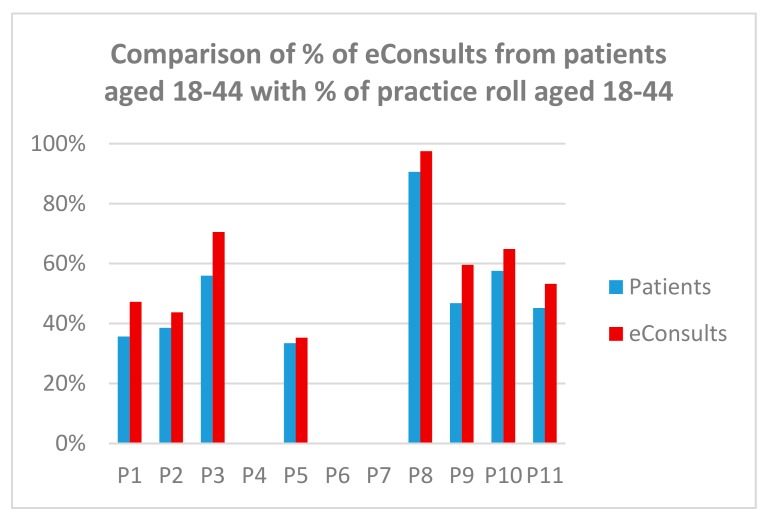
Comparison of the percentage of the eConsult submissions from patients aged 18–44 with the percentage of adult patients in that age group in the trial practices (excluding P04, P06 and P07).

Figure 7 shows that younger patients (18–44 years) submit proportionately more eConsults: 64% of eConsults were submitted by patients in this age group, whereas this age group accounted for 51% of patients aged over 18 in the trial practices.

#### 3.1.4. Deprivation Profile in the eConsult Trial

**Figure 8:**
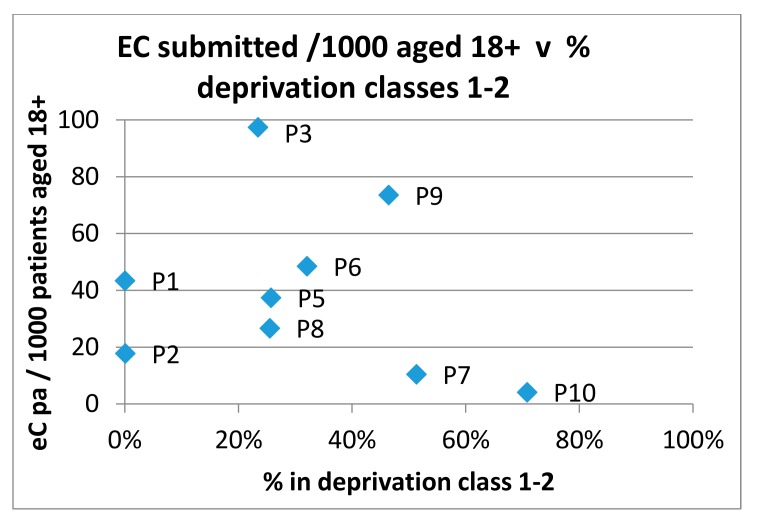
eConsult submission rates versus percentage of patients in the most deprived quintiles.

The submission rate of eConsults was compared to the percentage of patients in quintiles 1 and 2 (most deprived) according to the Scottish Index of Multiple Deprivation (SIMD) [6] for each practice in the trial. The results are shown in Figure 8.

**Figure 9:**
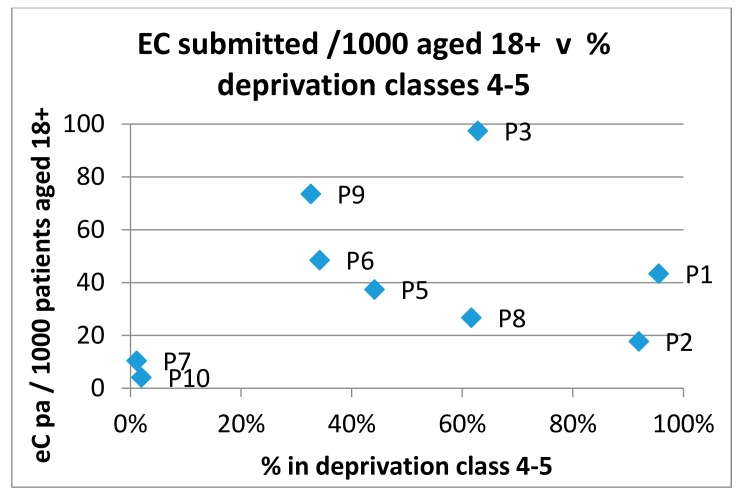
eConsult submission rates versus percentage of patients in the most affluent quintiles.

Similarly, Figure 9 shows a comparison of submission rates to the percentage of patients in Quintiles 4 and 5 (least deprived). The figures show there to be no discernable relationship between eConsult submission rates and measures of deprivation.

### 3.2. Patient Opinion and Surgery Staff’s Perceived Patient Opinion of eConsult

Patient surveys were complete by 6.5% of eConsult users. Patient survey data indicated strong satisfaction with eConsult (91.4%), with 91.5% of respondents likely to recommend eConsult to others. Patient survey free text comments were combined with the data arising from the GP staff interviews and using a thematic framework approach the following key themes emerged from the data:

• Flexibility around eConsult use

There was consensus around the flexibility gained from eConsult use, with patients greatly appreciating how the service could fit around them and their lifestyle.

*“As someone who works 9–5 it is very convenient service. It is trustworthy and reliable which makes it even better.”*

*(Patient)*

*“Sometimes you see it through the night, like, when they’ve finished work, seven o’clock or eight o’clock at night you can see the times, we get them the next morning so when they finish their work that’s when they’re doing the eConsults.”*

*(P6_Admin)*

*“…sometimes you can be on the phone twenty plus minutes waiting to get through on the triage line because it is so busy, so it certainly saves a lot of people some time, especially people who are at work who maybe can’t necessarily do a phone call but can quickly jump on the internet and submit an eConsult online.”*

*(P4_PM)*

• Flexibility around how to communicate with GP

Patients and GP staff commented on the benefits of providing individuals with different means of communicating with their GP. This flexibility was felt to ensure that patients felt they had a means of communicating in a way that suited them and they were comfortable with:

*“Don’t feel rushed or that I am bothering a GP with maybe trivia. Able to get the point across especially if one can tend to be ‘long-winded’”*

*(Patient)*

*“It was to do with depression or something, the clinician felt that the eConsult had actually helped because they didn’t feel that the patient would’ve come in for a face to face consultation…”*

*(P7_Admin)*

*“… is you know, how d’you book your holiday and how d’you do your shopping, and then it shows you a picture of a GP surgery in the early twentieth century and a GP surgery at the start of the 21st century and it’s the same, it’s just a pile of folk sitting in seats waiting to be seen by the doctor and that’s true. So I get the fact that this is another way of consulting”*

*(P9_GP)*

*“People maybe prefer that interaction with a human being…and people may have difficulty in describing what’s wrong with them, you know, putting that down on paper when perhaps it’s easier for them to verbalise it”*

*(P7_Admin)*

• Issues concerning when eConsult is appropriate to use

There were some concerns raised around when eConsult should be used and when other means of communication were more appropriate. It was felt that given the length of time taken to enter the required details for an eConsult, more clarity should be provided as to whether the eConsult was appropriate to complete for the patient’s current need.

*“For some people they might just think ‘four pages cause I’ve got hay fever, it’ll be easier, I’ll just go and get an appointment at the doctor’”*

*(P2_PM)*

*“Using the system for the first time, found it a bit frustrating repeating answers to some of the questions.”*

*(Patient)*

However, such comments were balanced out by patients who felt the service suited their needs and sat well with existing services:

*“The response was very quick, the advice I was given was what I was looking for and although I felt I didn’t need a face to face appointment, I had one as requested by the doctor. It is a good service to have especially if you feel you don’t want to waste time taking a valuable appointment when it may not be necessary to see someone face to face”*

*(Patient)*

### 3.3. Impact of eConsult on GP Surgery and Staff

The data arising from the semi-structured interviews and focus group were coded and analysed. The following themes around the impact of eConsult on the surgery and its staff were identified:

• Clinical decision making

GPs interviewed were very confident in patients using eConsult and did not feel that its use posed any risk to patients.

*“Yeah we had a look at it clinically before we sort of went into it really and looked at a few, we did the test site so you could look at that, so yeah it seemed quite sensible and appropriate red flags and things like that. So we haven’t any particular clinical concerns about it.”*

*(P5_GP)*

In addition, they felt it complemented the existing services offered, and in some cases, enhanced the face-to-face consultation through provision of information prior to meeting with the patient.

*“There’s a definite benefit to seeing information all in one go rather than hearing it on a phone call of course you’re trying to remember what people are saying and you’re taking notes at the same time and you’re trying to think what you’re going to ask them and all that, and that can be a bit confusing.”*

*(P1_GP1)*

*“There was one who had a works medical examination and something wasn’t quite right with some of the blood tests that they did and so he sent an eConsult to say ‘they told me this, can you give me some advice on it?’ and the advice was ‘obviously that gives us a bit of a head start, I’ll write you a form to get some other bloods done just to further investigate that and then we can discuss the results at a later date’ so that helped the process.”*

*(P6_GP)*

• Issues with fitting eConsults into existing practice processes

Each practice adopted slightly different ways of embedding eConsults into daily practice, some more successfully than others. As the number of eConsults each practice received per week was relatively low, there were concerns raised around eConsultations overlooked, or not being dealt with in a time-appropriate manner.

*“the one that was particularly unfavourable for us was one who was distressed that we didn’t get back in the time span and we didn’t give them what they wanted etc., however it was one of those that didn’t pick up the phone and we had emailed them, but they hadn’t got back to us sort of thing”*

*(P8_PM)*

*“like one today, like a UTI, one of the doctors says ‘it’s a UTI, needs prescription’ but because, like, from a Friday morning we don’t have to reply until end of Monday but she’ll need medication today, you know what I mean, so if we didn’t hurry things through, like, we normally say ‘oh an eConsult’s come, we better deal with it now’, if we did just follow the timescale she’d be all weekend without a prescription.”*

*(P1_Admin)*

*“I was jumping from one thing to the next that I could be just about ready to go home and think ‘oh my goodness there was three eConsults today and I’ve not emailed them back’, so I didn’t like that, maybe I’m just a bit forgetful but I just felt that it wasn’t safe.”*

*(P9_GP)*

At the current low volume in most practices, there had been little need to consider how to fully integrate eConsults into current processes and this had led in some cases to a perception of eConsults creating extra work. However, this was counterbalanced by the recognition that an eConsult would have been replaced by an alternative type of contact, with the same outcome. Perceptions of whether eConsult added or reduced the administrative workload were often closely related to the systems in place for dealing with submissions. Where these differed little from normal processes for booking appointments or dealing with other clinical processes, the potential for reducing the volume and length of telephone calls was clearly recognised.

One of the most significant impacts on the administrative workload in the implementation of eConsult is contacting patients to inform them of the outcome of the consultation, in particular to ask them to arrange an appointment. Most practices appeared to make at least three attempts to phone the patient, frequently reporting that contact could not be made. Some expressed a concern that patients would not respond to a withheld number, despite the notification on eConsult that calls may be made on such a number. This differed from processes in place to manage telephone consultations, where patients could be given a close estimate of the time they would be called and expressly informed that it would be from an unavailable number. While some practices had evolved methods to facilitate email contact, there remained a degree of concern about data security, particularly around shared mobile phones and email addresses. As a result, little more than basic information was transmitted.

Concerns were also expressed around integration of eConsults into the appointment system, and whether they may impact on the availability of urgent appointments for other patients. This was often perceived as “skipping the queue”, an impression that was reinforced by some of the eConsult marketing material.

### 3.4. Time Implications Associated with eConsult

A variation in processes between practices, and the way in which eConsults were handled resulted in significant variation regarding time spent on eConsults by different staff. In addition, there was further variability in assessment of the time spent on an eConsult in comparison to taking a phone call. Consensus was that a straightforward phone call simply requesting an appointment could be handled quickly and in less time than processing an eConsult. There was recognition, however, that where receptionists needed to assess whether an urgent or routine appointment was required, phone calls could take more time than an eConsult.

Similarly, there was no fixed pattern in terms of time impacts for GPs. Most practices operate on the basis of 10-min face-to-face appointments and five minutes for telephone consultations where these are offered. Where eConsults were slotted into appointment times, they generally replaced a five-minute telephone consultation. One of the practices with a relatively high volume of eConsults had calculated that each eConsult took approximately 2.5 min to assess and thus, two eConsults could be fitted into the time allocated for telephone consultations, thus potentially offering a significant time-saving.

Estimates were made for the time required for administrative staff to complete their normal booking and eConsult tasks (see Table 2). Time estimates were also made for GP eConsult processing times (see Table 2). These estimates were based on times provided in the interviews. The main variation in eConsult processing timings was due to the amount of patients’ notes consultations required. Estimates have been made based on half of eConsults requiring detailed use of patients’ notes and half requiring minimal use of notes.

Timings for administrative and GP time were estimated for a range of types of eConsult (see Table 3). These were weighted by the likelihood of that type of eConsult. The weights were based on eConsult data analysis provided by practice P2 which indicated that 43% of eConsults resulted in a further appointment (GP consultation; GP phone consultation or an appointment with a nurse/ANP), 27% of eConsults were administrative, and the remaining eConsults either required a prescription or no further action from the GP. The data provided by Practice 2 did not give precise percentages for type of appointment, administrative task, etc., but these were estimated from information provided in the interviews. The resulting timings are shown in the bottom row of Table 3.

eConsult submissions can either result in a GP appointment, replace a GP appointment or be the type of query that would be dealt with by administrative staff (e.g., a change of details). The pilots in England and Scotland have not collected sufficiently detailed information for accurate estimates of these types of queries. However, the pilot in England estimated that one eConsult would save 0.6 of a conventional GP appointment. This was based on 40% of eConsults generating a further face-to-face appointment with the GP [25].

The current evaluation found that practice in Scotland appears to be different. The categories of eConsult which would have previously required a GP appointment are: fit note; GP no action (e.g., follow-up); GP prescription; other appointment e.g., bloods; and GP phone back. These are estimated to account for 73% of the eConsults submitted. In addition, some patients who submitted eConsults would not have sought help if eConsult had not been available. This is estimated to be up to 8% of patients surveyed in England [25]. Hence, it can be assumed that between 67% and 73% (67% = 73% × 92%) of conventional appointments could be saved by using eConsult.

### 3.5. Cost/Effectiveness of eConsult

eConsult was not perceived as adding to existing surgery costs, as marketing and implementation costs were covered by the pilot programme. However, at the current levels of submissions, there was a general consensus that eConsult did not offer cost savings, as it was not possible to assess whether it was having any beneficial impact on reducing the number of face-to-face appointments or other services. As such, there would be little economic impetus to adopt the service in the short term.

*“at the moment it’s a service that a small number of patients are using and it’s an avenue for them and it’s not huge numbers, it’s picking up but it’s maybe one a day or two a day or something, you know, so for us it’s not saving us much time at the moment, you know, so there’d be no... in us investing in it, we wouldn’t get much back for it. It’s more like a... as things in time it might become more useful and more of an added bonus but right now it’d be hard to think how we would, how we could justify paying for it when it’s not giving us much back.”*

*(P1_GP2)*

A cost analysis was conducted to address potential savings associated with eConsult use. Costs of £95.08 per hour for GP time and £17.82 per hour for administrator time have been used in this evaluation. A detailed derivation of these costs is provided in Appendix B. Using these costs and the eConsult time estimates presented in Table 3, it is possible to calculate the total cost savings per eConsult submitted. Table 4 shows the cost savings, assuming different percentage savings of GP appointments per eConsult submitted.

**Figure 10:**
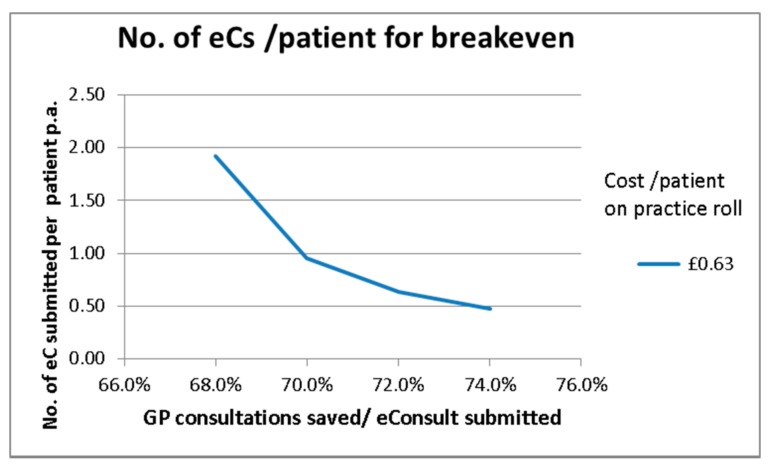
The number of eConsults /patient required versus % of consultations saved for breakeven.

It has been estimated that an eConsult will save somewhere between 0.6 and 0.74 of a GP appointment. This figure was established from data collected in this trial and is similar to the data collected in the English pilot [11]. As an eConsult takes more administrative time than a traditional appointment, the cost saving may be negative (Column 2, Table 4). However, as the GP time for dealing with an eConsult is on average less than that for a traditional appointment, there will be a GP cost saving per eConsult submitted (Column 3, Table 4). As the GP cost saving is greater than the additional administrative cost, there will be a net cost saving per eConsult submitted (Column 4, Table 4). This cost saving has then been compared to the annual charge per patient likely to be charged (by the Hurley Group [12]) for using the eConsult service. Hence, the number of eConsults submitted per patient that would be required to generate sufficient savings to cover the annual eConsult charge per patient can be calculated; this is shown in Figure 10. If a cost of £0.63 per patient per year is used, a maximum 73% of GP consultations can be saved per eConsult submitted, then a submission rate of just over 0.5 eConsults per annum per patient is required for breakeven (the average submission rate in the Scottish pilot was 0.05 per patient with a maximum rate of 0.13 being recorded at practice P3 in August 2017, see Appendix C). If the percentage of GP appointments saved is lower, then higher submission rates are required for breakeven.

## 4. Discussion

### 4.1. Summary of Findings

eConsult was perceived to offer a safe and effective service, but the level of effectiveness was felt difficult to assess given low numbers and a large variation in surgery expectations of the system. Despite this, staff felt the system complemented existing services but there was concern raised around how best to integrate its use into current practice processes. Patients’ perception of eConsult was generally positive, with the ability to use it anytime and the option of an alternative way of communicating with their GP seen as highly beneficial aspects of the system. However, it was felt that more direction could be provided as to the types of conditions/requirements most suited to completion of an eConsult. Clinicians were confident in their patients using eConsult and felt the information provided was trustworthy and appropriate. The potential for eConsult to save time and costs were apparent, both for the patient and the GP surgery.

The study also sought to identify aspects around the implementation of eConsult that were seen to support or hinder the introduction and integration of the system. It was evident that factors which facilitated the implementation of eConsult in the practices were: the presence of a ‘champion’ who could address issues and promote engagement; use of innovative methods to promote appropriate use of the system; and engagement of staff in all areas of the practice. Barriers to implementation included: delays in system start-ups due to strategic decision-making processes, leading to loss of engagement; marketing not being aligned with practice expectations of eConsult; challenges in integrating eConsult with existing systems; low levels of sharing experience and good practice; low volumes of eConsults leading to some frustration and inability to assess effectiveness; and inconsistencies in eConsult processing. Addressing both the themes arising from the interviews and these perceptions around barriers and facilitators to successful implementation of eConsult, in the next section we detail recommendations for future roll-out of digital eConsultation systems.

### 4.2. Recommendations for Roll-Out at Scale

Conducting the eConsult evaluation has allowed us to identify and better understand some of the key drivers in supporting/hindering successful implementation of the tool. In order to maximise the potential for successful implementation of similar digital consultation and self-care advice tools in the future, it is important to address these drivers, and tailor future strategic roll-out accordingly. The following sections detail our recommendations for measures to adopt in rolling-out similar systems in the future.

#### 4.2.1. Adoption of an Implementation Framework

In recent years, this lack of evidence around successful implementation has been recognised and researchers in the field have proposed key models, theories and frameworks designed to support the implementation of initiatives across health services. These frameworks seek to provide a mechanism by which successful introduction of an intervention into practice is optimised by addressing factors such as current practice, context and current processes and how these may impact on the successful integration of a new initiative [26,27,28]. Through use of a framework, an assessment as to the context into which a digital tool is delivered, and formulation of an appropriate plan of implementation (both initial delivery and to encourage embedded use) can be performed.

#### 4.2.2. Clarify Objectives of Adopting a Digital Tool

Prior to implementation, we would recommend facilitated discussion with key practice staff around their expectations of any digital tool and what the practice perceives as the main reasons for its adoption. Example areas such as discussion should focus on including: What does the practice regard as the main purpose for using the digital tool? Is its use to be promoted to all or a specific target patient group within registered users (for example, patients with specific conditions, patients frequently requiring repeat prescriptions)? What does a “success” outcome look like? Does success equate to a specific proportion of a certain patient group regularly using the system or perhaps to an overall proportion of registered users regularly using the digital tool?

#### 4.2.3. Understand the Digital Tool’s Fit with Current Practice Processes

Prior to implementation, it is essential that a clear understanding of how the practice currently operates is obtained. We would recommend construction of practice process diagrams that outline how the practice currently operates. Along with clarification of objectives, it is then important that how these objectives can be achieved in a way that fits well with current provision of services is explored.

#### 4.2.4. Strategic Roll-Out

Having spent time pre-implementation understanding current practice, roles and processes, and deciding upon how successful implementation of a digital tool for advice and consultation might look, it is important to be strategic in how the system is implemented. The following 3-stage implementation strategy is proposed by way of example:

- Stage 1—early implementation: A clear strategy is put in place for introducing the digital tool to the practice. This needs to include plans for staff engagement and education, patient engagement and education, and discussions with the practice manager and/or local champions to discuss the best way of introducing the digital tool. As part of this stage, a proportion of registered users might be targeted to trial the digital tool, receiving appropriate patient education and marketing and given opportunity (perhaps via Patient Public Involvement (PPI) workshops) to provide feedback on their experience. Feedback from practice staff should also be sought and any necessary refinements to process made.
- Stage 2—growth of digital tool usage: Having introduced the digital tool in stage 1 and adopted processes to fit with practice and patient needs, the tool is introduced to a wider population within the registered practice users. Again, patient education is provided and appropriate marketing of the tool provided to ensure a clear vision for how the digital tool should be used and how it integrates with existing practice services. In addition, work around how to sustain use of the digital tool is conducted, consulting with patients and staff to ensure that its use meets patients’ needs and expectations and fits well with the care provision model of the practice.
- Stage 3—deployment of the digital tool at scale: The final stage of implementation is focused on sustained, successful integration. This stage focuses on successful implementation and how this manifests itself for a given GP surgery. Targets are reviewed in terms of patient population using the digital tool, time and cost savings achieved, and how well the system has become embedded in day-to-day practice. Evaluation of the digital tool should address patient satisfaction with the service, surgery satisfaction with the service as well as time and cost saving factors associated with its use. In addition, the digital tool should be evaluated against the Health Quality dimensions [29] as well as key Government strategy documents to assess its contribution to patient-related care measures.

#### 4.2.5. Implementation Partnership

Data from the interviews suggests that practices would have welcomed more support during implementation, along with personalized strategies to assist with integration of eConsult with existing processes. We would suggest that prior to implementing a digital tool, an Implementation Partnership is formed comprising of site champions (a key member of staff from both the clinical and administrative staff teams who will promote the digital tool) and an external support facilitator (a member of the Scottish Government team for example). This Implementation Partnership is then pivotal in the roll-out of the digital tool through the 3 stages outlined above, offering the potential to liaise on any issues, and greater consistency in the case of any staff changes.

#### 4.2.6. Focused Marketing Efforts

Rather than directing all patients to the digital tool regardless of why they are contacting the surgery, analysis of the data would suggest that a better use of the service would be to ensure that use is promoted to certain individuals, for specific requirements. This could include, for example, patients: requiring follow-up appointments; with general administrative queries; requiring repeat prescriptions; or seeking general advice. This directed marketing would ensure that patients who will benefit most from using the digital tool are encouraged to use the service and those where a face-to-face/phone appointment is likely to be the outcome are directed to calling the surgery. Such an approach is in line with the 2020 Vision of ensuring right patient, right time, right place, right team, every time [30].

By targeting the use of digital consultations to patients, it is possible to direct use to the types of consultation that are likely to save a GP appointment. We have analysed the savings associated with different percentages of patients requiring a follow-on GP appointment in order to show the potential savings involved with directed marketing. These are shown in Table 5.

The base case is the current situation where no directed marketing occurs. The four scenarios illustrate a move to a range of digital consultations which would not require a further GP appointment, this would be achieved through targeted marketing. The shaded cells show the digital consultation types which could replace a traditional appointment. In Table 6, the appointments saved reflect the total of the shaded cells for that scenario as shown in Table 5. The minimum appointments saved refers to the previous figure adjusted to take into account findings from the English pilot study which found that 8% of eConsults submitted reflect the circumstance where the patient would not otherwise have attempted to consult their GP.

The aim of Scenarios 1–4 is to show how efficiencies can be achieved if the patient is directed to use eConsult in situations where the electronic consultation is most likely to replace an appointment. In Scenario 1, patients are directed to use eConsult for fast access to fit notes and discouraged from use where a physical examination may be required e.g., dermatological conditions. In scenarios 2–4, the use of eConsult to replace a follow-up appointment is encouraged, where the patient informs the GP of successful outcome of current treatment (unsuccessful treatment may require an appointment). In Scenario 4, eConsult is encouraged for the re-issue of previously prescribed medicines (not repeat prescriptions).

**Figure 11:**
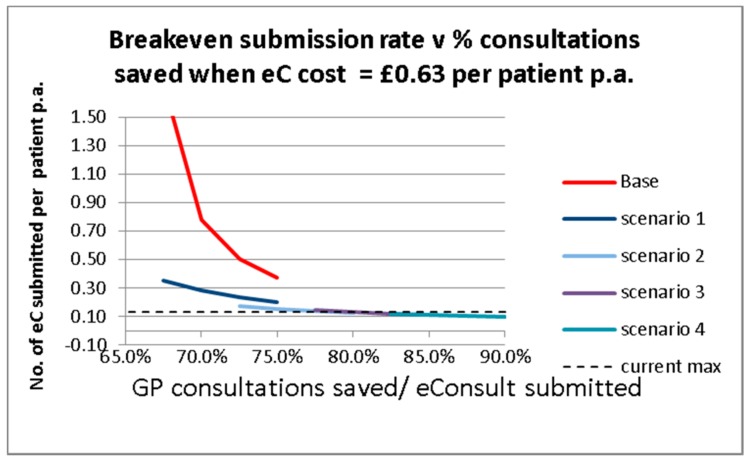
eConsult submission rates required for breakeven at a cost of £1 per patient.

Figure 11 shows the digital consultation submission rates per patient per annum required, assuming an annual cost per patient of £0.63. In August 2017, practice P3 achieved a submission rate of 13 per patient, hence Scenarios 3 and 4 break even with usage rates that have been achieved in the pilot study.

#### 4.2.7. Patient Engagement and Education

We would recommend focused effort on patient education regarding provision of services. At a national level, this needs greater marketing around the changing face of primary care provision, and promoting the partnership between health and social care services in line with the Scottish Government’s strategies around this. At a local level, it is important to engage registered surgery users in dialogue and discussion around service provision and ensure that patients feel part of any changes promoted. It is important that patients are comfortable with any proposed changes, are educated in how a digital tool for advice and consultation integrates with current service provision and feel able to provide feedback on thoughts/suggestions/concerns once the service is in place. It is recommended that patient participation groups would provide a useful mechanism to facilitate this.

#### 4.2.8. Staff Engagement and Training

It is important that all practice staff engage with the digital tool and understand how its use integrates with current practice processes. We would advise that the Implementation Partnership work with practice staff to ensure that at all stages of implementation, staff receive consistent information on factors affecting the implementation, training as to how the digital tool will operate, and are made aware of individual responsibilities and changes in processes adopted. Regular meetings should be held through all stages of implementation both to educate, and to receive and act on feedback.

#### 4.2.9. Implementation Support Network

Further exploration of how a network supporting individual practices adopting a digital tool is recommended, for example, a network website where different aspects of implementation are addressed such as marketing, staff engagement, patient involvement, and ideas to support and facilitate these. Such network support, together with the Implementation Partnership, may help practices feel less isolated in their adoption of a digital tool and more able to share experiences and learn from each other. In addition, the cluster GP model [31] may be a further way of facilitating this.

#### 4.2.10. Digital Advice and Consultation Tool Infrastructure

Reflecting on the data analysed, it would seem pertinent to ensure that any system selected is refined to accommodate the requirements outlined in order that the service meets the needs of both patients and practices alike. It is particularly important that concerns are addressed with regards to patient safety and the potential for litigation if patient contact is not made within an appropriate timescale for an urgent condition. These include:

- The ability to provide locally relevant content. This could include, for example, signposting to local services or linking to other cluster GP practices.
- The ability to tailor marketing such that it is clear to patients what the tool provides and in what circumstances (for example, what conditions, situations) might it be recommended for use.
- The ability to tailor response times dependent on the condition such that an appropriate timely response can be assured.

With respect to any future evaluation, it would be pertinent to consider whether more detailed information as to how the digital tool is used by individuals could be provided. For example, being able to map an individual visit to the digital tool in terms of pages accessed, time stamp information and sequence of navigation.

### 4.3. Strengths of the Study

The study is the first to evaluate eConsult use across Scotland. It provides an insight into the experiences and challenges of implementing electronic self-management and consultation tools in a primary care setting across diverse GP settings.

The data collected over the 6-month evaluation has provided significant insight into how eConsult has been implemented across the 11 pilot practices. Undertaking the evaluation at this relatively early stage in the implementation process minimised recall bias of issues relating to the introduction of eConsult and its integration with existing systems, initial training and marketing. It has also highlighted areas of immediate concern which may affect the willingness to adopt or retain eConsult or a similar system. This will allow processes to support future implementation of such a system to be developed in advance and inform future enhancements of eConsult.

Results are comparable to those found by an evaluation of eConsult in NHS England [9,11]. Findings from this study focused on how eConsult impacted on clinical decision-making; GP workload and staff’s perception of patients’ experience of eConsult. As with our evaluation, results suggest that eConsult was felt useful for specific conditions and types of consultation, that workload was not decreased and, in general, patients that used eConsult were felt to benefit from the service.

### 4.4. Study Limitations

As the evaluation was only conducted over a 6-month period, it was not possible to assess the long-term sustainability of eConsult processes adopted. In addition, the qualitative evaluation in particular (interviews with GP surgery staff) only provided a snapshot of current thoughts around usage of eConsult. Such an evaluation can be limited as thoughts/comments provided are skewed to views on eConsult that week/month/couple of months. In order to ensure that a full picture of how eConsult has been perceived throughout the period of its use, a longer-term study is required, where interviews with staff are repeated at appropriate time intervals.

The data from the patient survey and the eConsult log was provided to us by the Hurley Group and some of the specifics around the data were unavailable to use. For example, data relating to patients who accessed eConsult but did not go on to complete a consultation (perhaps because their needs were met by the self-help information) was not available to us. This meant that the full effectiveness of eConsult could not be assessed. In addition, regarding the patient survey, we had no input into the questions asked as these were decided solely by the Hurley group. In addition, it is entirely possible that patients completed a patient survey more than once and we were unaware of this. It would have been preferable if unique patients/repeat patients were identifiable.

Lastly, due to the time constraints of the project, the research team was unable to interview patients directly about their eConsult experience and were restricted to obtaining patients’ views from the patient survey data. As this was only completed by a minority of patients (6.5%) it is entirely reasonable that the views expressed in the survey are not representative of the wider population. A more in-depth longitudinal evaluation of eConsult would provide us with an opportunity to better understand how to fully integrate eConsult into primary care practice, and explore further how the system is received and used by patients.

### 4.5. Comparison with the Literature

Few studies have been conducted around use of digital advice and consultation tools for primary care provision [9]. However, our findings would seem to emulate those found in previous studies. Our findings specific to eConsult largely mirror those presented by the English evaluation of eConsult, with small differences in some of the cost/effectiveness figures due to differences in the health care models [11]. Our recommendations around patient education, tailoring of eConsult to specific users and/or conditions are reflected in the existing literature [26,32,33]. With regard to issues around integration with existing practice processes and impact on workload, Chang et al. [10] found that clear guidance around how to ensure successful adoption and integration of digital systems is key. Lastly, the general consensus that the introduction of digital services to support primary care provision is a positive step replicates the findings of Liddy et al. [7], who conducted a similar study around the use of Champlain BASE (an e-Consultation tool for primary care). Their findings report that primary care providers had high satisfaction with the digital eConsultation service and felt perceived benefits included provision of high quality of care and appropriate information provided in a timely manner. This finding is also mirrored by Vimalanda et al. [34]. Their research systematically reviewed the literature on use of eConsultations to improve access to specialty care, and from the 27 articles reviewed reported high satisfaction rates amongst primary care providers.

## 5. Conclusions

Through this evaluation of eConsult across Scotland, the tool has been shown to have the potential to significantly impact on the provision of primary care services and the way service users interact with these services. In addition, the study has identified key recommendations for successful implementation of a digital advice and consultation tool. Consideration also needs to be given as to how such a tool is integrated into future digital care provision to ensure that patient care using digital services is cohesive and seen to enhance current service provision. Lastly, in order to ensure that any digital tool is aligned to the HealthCare Quality Dimensions for Scotland [29], it must address concerns relating to the timeliness of the response a patient receives.

## Figures and Tables

**Table 1:** Complement of staff interviewed across 11 GP practices.

GP Practice	Interview/Focus Group	Staff Involved
P1	Interviews	Practice Manager, 2×GP, Pharmacist, 3×Admin
P2	Interview	Practice Manager, 2×GP, Admin
P3	Interviews	Practice Manager, Assistant Practice Manager, 2×GP, 2×Clinical Coordinator
P4	Interviews	Practice Manager, GP, Admin
P5	Focus group	Admin, Practice Manager, Senior Receptionist, GP
P6	Interviews	Practice Manager, 2×GP, 2×Admin
P7	Interviews	2×Admin, 4×GPs, 2×ANPs
P8	Interviews	GP, Practice Manager
P9	Interviews	Practice Manager, GP, Admin
P10	Interview	Practice Manager, Nurse Practitioner
P11	Interviews	Practice Manager, GP, 2×Admin
Total		48 interviews

**Table 2:** Estimated timings for eConsult related tasks.

**Administration Activity**	**Expected Time (Min)**
Book normal GP appointment	3
Receive eConsult	3
Close off (including contacting patient)	3
Close off (no patient contact)	1.5
**GP Activity**	
Face-to-Face appointment	10
Telephone GP appointment	5
Process an eConsult with minimal use of patient notes	2.5
Process an eConsult with detailed use of patient notes	5
Generate a sick note (e.g., extension to sick leave)	4
Generate a prescription	Note check + 1
Other: e.g., generate another appointment, bloods, nurse.	Note check + 0.5
Change of details	0.5

**Table 3:** Timings for different types of eConsult.

eConsult Type	Estimated % of eConsults Received	Admin Time per eConsult (min)	GP Time per eConsult (min)
eC admin: other & details change	12%	4.50	0.50
eC admin: fit note	15%	6.00	4.00
GP no action (e.g., follow-up)	10%	4.50	3.75
GP prescription	20%	6.00	4.75
Other appointment e.g., bloods	13%	6.00	4.25
GP phone back	15%	6.00	3.75 + 5
GP appointment	15%	6.00	3.75 + 10
Total expected time per eConsult	100%	5.7	5.9

**Table 4:** Cost savings for different assumptions of %GP appointments saved per eConsult submitted.

%GP Appointments Saved per eC Submitted	Admin Staff Cost Saving (£/eC)	GP Cost Saving (£/eC)	Total Cost Saving (£/eC)
74%	−1.0	2.4	1.3
72%	−1.0	2.0	1.0
70%	−1.1	1.7	0.7
68%	−1.1	1.4	0.3
66%	−1.1	1.1	0.0
64%	−1.1	0.8	−0.3
62%	−1.1	0.5	−0.7
60%	−1.1	0.1	−1.0

**Table 5:** Types of eConsult currently submitted and assumed in the 4 future scenarios (NB. Patient recall refers to eConsults that result in the patient returning to the surgery).

eConsult Type	Base 27% Admin 43% Patient Recall	Scenario 1 40% Admin 30% Patient Recall	Scenario 2 35% Admin 25% Patient Recall	Scenario 3 30% Admin 20% Patient Recall	Scenario 4 20% Admin 15% Patient Recall
eC admin: Change of details & other	12%	15%	15%	10%	5%
eC admin: Fit note	15%	25%	20%	20%	15%
GP no action (follow-up)	10%	10%	20%	30%	40%
GP prescription	20%	20%	20%	20%	25%
Other appoint. e.g., bloods	13%	10%	10%	5%	5%
GP phone back	15%	10%	10%	10%	5%
GP appointment	15%	10%	5%	5%	5%
Total	100%	100%	100%	100%	100%

**Table 6:** Potential GP appointments saved, current and in the 4 scenarios.

eConsult Savings	Base 27% Admin 43% Patient Recall	Scenario 1 40% Admin 30% Patient Recall	Scenario 2 35% Admin 25% Patient Recall	Scenario 3 30% Admin 20% Patient Recall	Scenario 4 20% Admin 15% Patient Recall
Appointments saved	73%	75%	80%	85%	90%
Min. appointments saved	67%	69%	74%	78%	83%

## Appendix A

### Patient Survey

1. Overall, how satisfied or dissatisfied were you with using the Consult Online service for your health assessment? (Very satisfied/Fairly satisfied/Neither satisfied nor dissatisfied/Fairly dissatisfied/Very dissatisfied)
2. How likely are you to recommend the Consult Online service to friends and family if they need similar care or advice? (Extremely likely/likely/neither likely nor unlikely/unlikely/Extremely unlikely)
3. Seven days after your Consult Online request, had the issue you consulted about resolved? (Completely resolved/improved/the same/worse/not applicable)
4. In the week after your Consult Online request, did you have contact with the GP practice or any other health service for the same problem? (Yes/No)
5. Were you contacted by the practice about your eConsult request in the stated timeline? (Yes/No, I was contacted late/No, I was not contacted at all)
6. How did you hear about the eConsult service? (My GP told me about it/Someone else from the GP practice told me about it/From the GP practice website/Another patient, family member or friend told me about it/From an internet search/I read about it/From a leaflet or promotional banner/Other—write in)
  Free text comments

## Appendix B

### Appendix B.1. Estimated Unit Costs for eConsult Comparisons

The cost comparisons consider three components:

1. Cost of GP time
2. Cost of administrator/clerical time
3. Annual cost of eConsult

It is assumed that any use of eConsult would not affect the requirement for facilities such as space or IT equipment though large-scale adoption could affect this requirement.

### Appendix B.2. Cost of GP Time

The main source of the employment costs [34] is based on data from England but the costs in Scotland are unlikely to be significantly different. The data describing GP working hours [35] also refers to experience in England.

**Table A1:** Annual GP costs 2015/2016 [34,36].

Cost of GP Time	England	Scotland	Inflated to 2016/2017
A. Net remuneration	£103,800	£91,400	£93,228
B. Practice expenses			
B.1 direct care staff	£23,082	£23,082	£23,544
B.2 admin & clerical staff	£37,673	£37,673	£38,426
B.3 office	£10,007	£10,007	£10,207
B.4 premises	£15,120	£15,120	£15,422
B.5 other	£16,099	£16,099	£16,421
B.6 travel	£1200	£1200	£1224
C. Qualifications	£41,188	£41,188	£42,012
D. Capital costs	£15,463	£15,463	£15,772
A + B + C + D =	£263,632	£251,232	£256,257
A + C =	£144,988	£132,588	£135,240

The only costs considered in the eConsult comparisons are A + C. There is some evidence [36] that GP costs are lower in Scotland than England. The mean “net remuneration” includes full-time and part-time General Practitioner Medical Services (GPMS) contractors. The number of hours available for various patient care activities was estimated, again using data from the Personal Social Services Research Unit (PSSRU) unit costs [34]. The mean hours per week are based on a survey that includes full-time and part-time GPs. Given that the mean “remuneration costs” had a similar mix of full time and part time, the hourly cost is a reasonable estimate.

**Table A2:** Cost per GP available hour.

Unit	England	Scotland	Inflated to 2016/17
hours per week	41.4	41.4	41.4
weeks p.a.	42	42	42
hours p.a.	1738.8	1738.8	1738.8
direct patient care	62.10%	62.10%	62.10%
indirect patient care	19.70%	19.70%	19.70%
general admin	8.40%	8.40%	8.40%
external meetings	3.50%	3.50%	3.50%
other	6.30%	6.30%	6.30%
total	100.00%	100.00%	100.00%
available time	81.80%	81.80%	81.80%
£/GP year	£144,988	£132,588	£135,240
£/GP available hour	£101.94	£93.22	£95.08

### Appendix B.3. Cost of Administrator/Clerical Time

The equivalent estimate for administrative and clerical staff has been constructed from the typical annual staff cost including oncosts and reflecting the average mix of such staff in GP practices = £37,673/1.36 = £27,700 p.a. (Table 10.3a, [34]). The working hours and availability data need to be confirmed.

**Table A3:** Cost per administrator/clerical available hour.

Unit	Costings
hours per week	37.5
weeks p.a.	44.5
h p.a.	1669
available time	95%
£/h	£17.47
Inflated to 2016/7	£17.82

### Appendix B.4. Annual Cost of eConsult

A cost of £0.63 per patient aged over 18 years on the practice roll was used throughout the analysis, this is the cost currently quoted by the Hurley Group [12].

## Appendix C

### eConsult Submission Rates

Table A4 shows the actual number of eConsults submitted in each of the trial practices, these have been used to estimate the annual number of submissions. The number of patients on the practice roll aged over 18 years has been estimated (from the published number of patients between 15 and 24). Hence, the eConsult submission rate per patient is calculated.

**Table A4:** eConsult submission rates.

GP Practice	eC Submitted April–August 2017	Estimated eC Annual Submissions	Estimated Practice Roll Aged > 18	Submission Rate/Patient
P1	184	442	10,171	0.04
P2	48	115	6483	0.02
P3	606	1454	14,927	0.10
P4	0	0	14,725 *	0.00
P5	190	456	12,186	0.04
P6	120	288	5939	0.05
P7	19	46	4368	0.01
P8	96	230	8632	0.03
P9	246	590	8026	0.07
P10	10	24	5809	0.00
P11	122	293	4006	0.07
Total	1519	3646	80,546	0.05

* Excluded from analysis.

Table A5 gives details of the peak eConsult submission recorded by practice P3 in August 2017.

**Table A5:** Peak eConsult submission rate.

GP Practice	eC Submitted April–August 2017	Estimated eC Annual Submissions	Estimated Practice Roll Aged > 18	Submission Rate/Patient
P3	157	1884	14,927	0.13
